# Germ cell desquamation-based testis regression in a seasonal breeder, the Egyptian long-eared hedgehog, *Hemiechinus auritus*

**DOI:** 10.1371/journal.pone.0204851

**Published:** 2018-10-04

**Authors:** Diaa Massoud, Miguel Lao-Pérez, Alicia Hurtado, Walied Abdo, Rogelio Palomino-Morales, Francisco David Carmona, Miguel Burgos, Rafael Jiménez, Francisco J. Barrionuevo

**Affiliations:** 1 Department of Zoology, Faculty of Science, Fayoum University, Fayoum, Egypt; 2 Departamento de Genética e Instituto de Biotecnología, Universidad de Granada, Granada, Spain; 3 Department of Pathology, Faculty of Veterinary Medicine, Kafr El Sheikh University, Kafr El Sheikh, Egypt; 4 Departamento de Bioquímica y Biología Molecular I, Universidad de Granada, Granada, Spain; University Hospital of Münster, GERMANY

## Abstract

Testes of seasonally breeding species experience a severe functional regression before the non-breeding period, which implies a substantial mass reduction due to massive germ-cell depletion. Two alternative mechanisms of seasonal germ-cell depletion have been described in mammals, apoptosis and desquamation (sloughing), but their prevalence has not been determined yet due to reduced number of species studied. We performed a morphological, hormonal, and molecular study of the mechanism of seasonal testicular regression in males of the Egyptian long eared-hedgehog (*Hemiechinus auritus*). Our results show that live, non-apoptotic, germ cells are massively depleted by desquamation during the testis regression process. This is concomitant with both decreased levels of serum testosterone and irregular distribution of the cell-adhesion molecules in the seminiferous epithelium. The inactive testes maintain some meiotic activity as meiosis onset is not halted and spermatocytes die by apoptosis at the pachytene stage. Our data support the notion that apoptosis is not the major testis regression effector in mammals. Instead, desquamation appears to be a common mechanism in this class.

## Introduction

In temperate areas of the Earth, animals reproduce when the environmental conditions are optimal to maximize growth rate and survival of newborns. In most species living at these latitudes, photoperiod is the cue controlling their reproductive rhythm. However, other factors, such as food availability, stress, and weather, can either modify or even overcome the influence of photoperiod. Environmental cues act by modulating the expression of the hormones released by the hypothalamic–pituitary–gonadal (HPG) axis. The levels of serum gonadotropins are lower in the non-breeding period, a fact that in males leads to a reduction of circulating testosterone, which is associated to spermatogenesis inhibition and, as a consequence, to the loss of the germinative epithelium and the subsequent reduction in testicular size and mass [1]. Interspecific differences have been reported regarding the condition in which the inactive testis is maintained after seasonal regression. In some species, meiotic activity is not completely abolished during the non-breeding season as meiosis onset continues and primary spermatocytes are subsequently depleted by apoptosis [2, 3]. In contrast, in other species meiosis onset is halted and the seminiferous tubules retain only Sertoli and spermatogonial cells [4, 5]. Massoud *et al*., [6] reported that southern and northern populations of the greater white‐toothed shrew, *Crocidura russula*, exhibit different seasonal breeding patterns in the Iberian Peninsula. In the north, reproduction occurs in summer and males undergo complete testis regression in winter. On the other hand, in the south reproduction takes place in winter and no testis regression occurs during the non-breeding season (females are not receptive in summer). The authors proposed that this isa case of adaptive lack of seasonal involution.

In addition to germ cells, Leydig cells also show species-specific differences in the regressed testis [7].

The mechanisms of testis regression are also variable among species. Most studies have pointed to apoptosis as the main cellular event responsible for testis regression [8, 9]. However, more recently, germ cell desquamation (sloughing) has been proposed as a new mechanism of testis regression, as reported for both the Iberian mole *Talpa occidentalis* [10] and the large hairy armadillo *Chaetophractus villosus* [11]. Dadhich *et al*. [10] proposed that low levels of intra-testicular testosterone deregulate the expression of the cell-adhesion molecules, leading to the loss of Sertoli-Sertoli and Sertoli-germ cell junctions, which results in the sloughing of the meiotic and post-meiotic germ cells placed in the adluminal compartment of the seminiferous tubules. Moreover, in a very recent study, González *et al*. [12] proposed a novel mechanism of testis regression in which the balance between apoptosis and autophagy regulates this process, as reported in the vizcacha, *Lagostumus maximus*.

Thus, analysing the circannual testicular dynamics in new mammalian species would help 1) to elucidate which mechanisms of testis regression are evolutionary conserved and 2) to estimate the inter-species variation existing in the process. The long eared-hedgehog, *Hemiechinus auritus*, is one of the six African species of spiny hedgehogs belonging to the family Erinaceidae. It is distributed in the coastal semi-desert areas of Libya and Egypt, in the Cypriot island, the Middle East, and Central Asia. *H*. *auritus* is a terrestrial nocturnal mammal that lies in burrows and prefers mesic habitats including gardens, olive groves, and cultivated areas [13]. This species has a circannual cycle of vital activity, showing its maximum in summer (with a peak in July), whereas most individuals hibernate in winter for periods of up to 40 days. This cycle is accompanied by annual body mass fluctuations, being maximal in summer and minimal in winter [14]. Consistently, *H*. *auritus* is a seasonal breeder. Available data indicate that, independently of the geographical location, hedgehogs of this species breed in late spring-summer and are inactive in late autumn and winter [14–18].

Here we analysed the circannual testicular changes occurring in the long-eared hedgehog from northwestern Egypt during its seasonal breeding cycle. Testicular histology was studied and the spatial gene-expression patterns of several somatic and germ cell types were determined. The incidence of apoptosis and the serum testosterone levels were also quantified in both sexually active and inactive hedgehogs.

## Material and methods

### Animals and tissue preparation

Twelve adult males of long-eared hedgehog were captured alive in Matrouh governorate (31° 21' N, 27° 14' E) in the northwestern part of Egypt, 400 km from Cairo. Two study groups were established: animals captured in summer (from May to August, N = 6) and those captured in winter (January and February, N = 4). We also collected two individuals in the period of testis regression, at the end of September. Juvenile males (N = 2), identified on the basis of extremely low testis mass (<200 mg), reduced seminiferous tubule diameter (<70 μm), and low body mass (<140 g), were excluded from the study. Testes and epididymides were collected, weighed, and fixed overnight in a 50× volume of Serra's fixative (100% ethanol, 40% formaldehyde, and glacial acetic acid in a proportion of 6:3:1, respectively). This study was carried out in strict accordance with the recommendations of the Guide for the Care and Use of Laboratory Animals of the National Institutes of Health. The capture and experimental protocols were approved by the Ethics Committee for Animal Experimentation of the Zoology Department, Faculty of Sciences, Fayoum University. The long-eared Hedgehog is categorized by the Egyptian Wildlife Protection System as (LR/lc), (Lower risk/ least concern), thus, it is not an endangered or protected species. Animals were captured by a professional hunter with permission from the Egyptian Federation of Wild Hunting. To minimize animal suffering, hedgehogs were captured by hand in their burrows and immediately transported to the laboratory where they were euthanised by C0_2_ inhalation.

### Histology and immunofluorescence

Testes were embedded in paraffin, sectioned (5 μm), mounted on polylysine-coated slides (VWR, Belgium), and stained with hematoxylin and eosin according to standard procedures for morphological analysis. Single and double immunofluorescences were performed as previously described [6]. **Table 1** summarizes the antibodies and working concentrations used in this study.

**Table 1:** Antibodies used in this study.

Gene product	Host species	Working dilution	References
Laminin	Rabbit	1:100	Sigma L9393
Smooth muscle alpha-actin	Mouse	1:100	Sigma A2547
Claudin 11	Rabbit	1:100	Santa Cruz Biotechnology, CA sc-25711
DMC1	Goat	1:100	Santa Cruz Biotechnology, CA sc-8973
PCNA	Mouse	1:100	Santa Cruz Biotechnology, CA sc-56
P450scc	Goat	1:100	Santa Cruz Biotechnology, CA sc-18043
SOX9	Rabbit	1:500	MERCK Millipore AB5535
Beta-Catenin	Mouse	1:100	Sigma C7082
N-Cadherin	Rat	1:1	Hybridoma bank
Connexin 43	Rabbit	1:10	Santa Cruz Biotechnology, CA sc-9059

### Analysis of apoptosis

Apoptosis was assessed with the terminal deoxynucleotidyl transferase deoxy-UTP-nick end labeling (TUNEL) assay, using the Fluorescent In Situ Cell Death Detection Kit (Roche ref. 11684795910), according to the manufacturer's instructions. In order to quantify the incidence of apoptosis, the total number of TUNEL^+^ cells were counted in a total of 100 seminiferous tubule sections of each animal.

### Morphometrics and statistics

The diameters of 30 transversely sectioned seminiferous tubules of different testis sections were measured as previously described [6]. Seminiferous tubule diameter (expressed in microns) as well as body and testis mass (expressed in grams and milligrams, respectively) are reported as mean ± standard deviation values. Since these groups of data fit a normal distribution, we used Student's *t*‐test to compare the respective means.

### Serum levels of testosterone

Fresh blood samples obtained from the *H*. *auritus* males included in this study were stored at 4°C overnight and centrifuged next morning at 6,000 rpm for 20 min at 4°C. The supernatant (serum) was then stored at -80°C until further use. Testosterone levels were measured using the chemiluminescent enzyme immunoassay method in an Immulite 2000 analyzer (Siemens Healthcare).

## Results

### Testes of *Hemiechinus auritus* remain sterile during the winter

**Fig 1:**
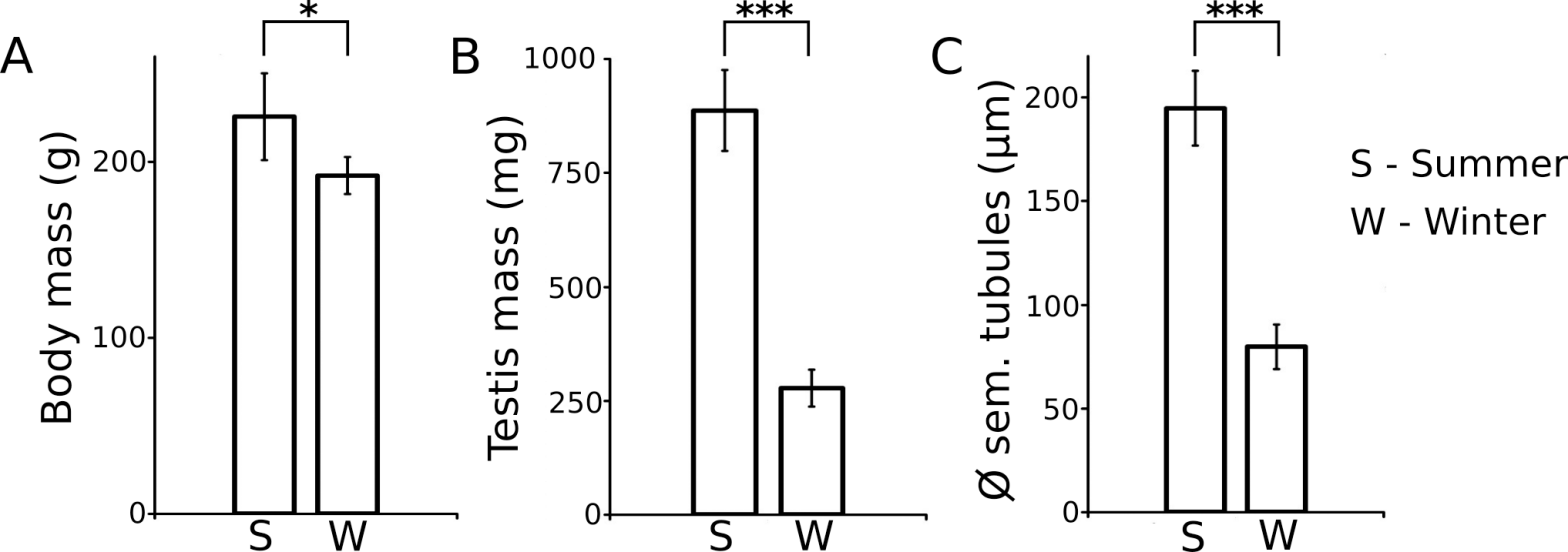
**Comparisons of three morphometric parameters between summer and winter groups of *H*. *auritus*:** (A) body mass, (B) testis mass and (C) seminiferous tubule diameter.

**Fig 2:**
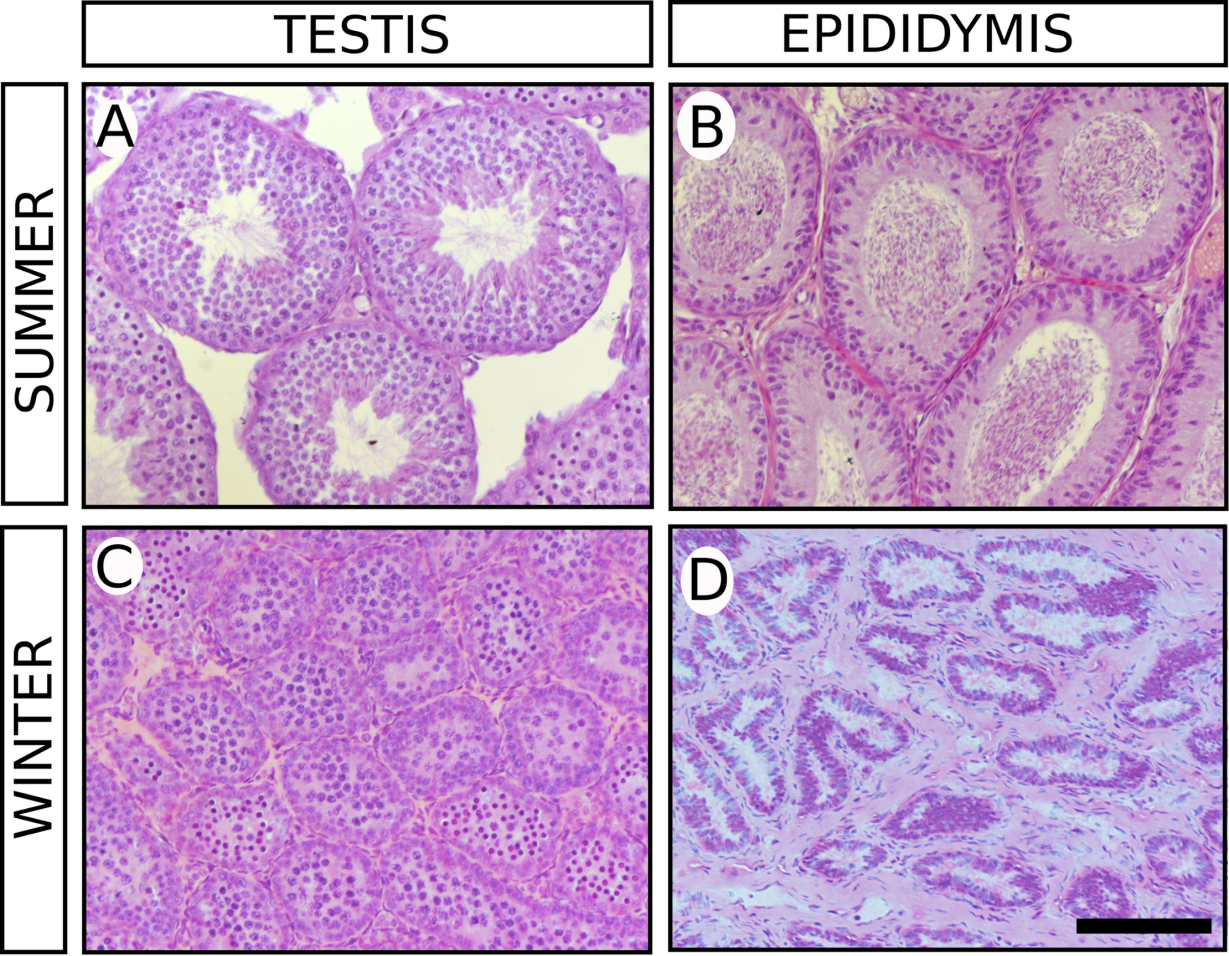
**Hematoxylin‐eosin‐stained histological sections of testes (A and C) and epididymides (B and D) from *H*. *auritus* males belonging to the summer (A and B) and winter (C and D) study groups**. In the summer group, the histology of the seminiferous tubules was the expected for sexually active males and the epididymides contained sperm. In contrast, testis tubules of animals collected in winter were reduced in size. They were filled with round cells resembling spermatocytes and no mature sperm was visible. In this season, the epididymal tubules are reduced in diameter and devoid of sperm. Scale bar shown in D represents 100 μm for all pictures.

Long-eared hedgehogs captured in summer were significantly larger than those from winter (summer body mass: 225 ± 24 g; winter body mass: 192 ± 10 g; two-tailed *t*-test, P = 0.019; **Fig 1A**). In the summer group, the mean testis mass was three times higher than that in the winter group (summer testis mass: 887 ± 88 g; winter testis mass: 277 ± 40 g; two-tailed *t*-test, P < 0.001; **Fig 1B**). At the histological level, testes in the summer group presented all features of an active and fertile testis, with seminiferous tubules in all stages of the spermatogenic cycle and spermiogenesis completed (**Fig 2A**). Accordingly, we found abundant sperm in the epididymides (**Fig 2B**). In contrast, winter testes showed marked reduction in seminiferous tubules (summer tubular diameter: 195 ± 18 μm; winter tubular diameter: 80 ± 11 μm; two-tailed *t*-test, P < 0.001; **Figs 1C and 2C**). In these tubules, no stage of the spermatogenic cycle could be identified and most of them were filled with primary spermatocytes, lacking secondary spermatocytes, spermatids, and sperm (**Fig 2C**). Consistently, the diameter of the epididymal tubule was reduced and empty (**Fig 2D**).

### Spermatogenesis is interrupted at the meiotic pachytene stage in the regressed testes of *Hemiechinus auritus*

**Fig 3:**
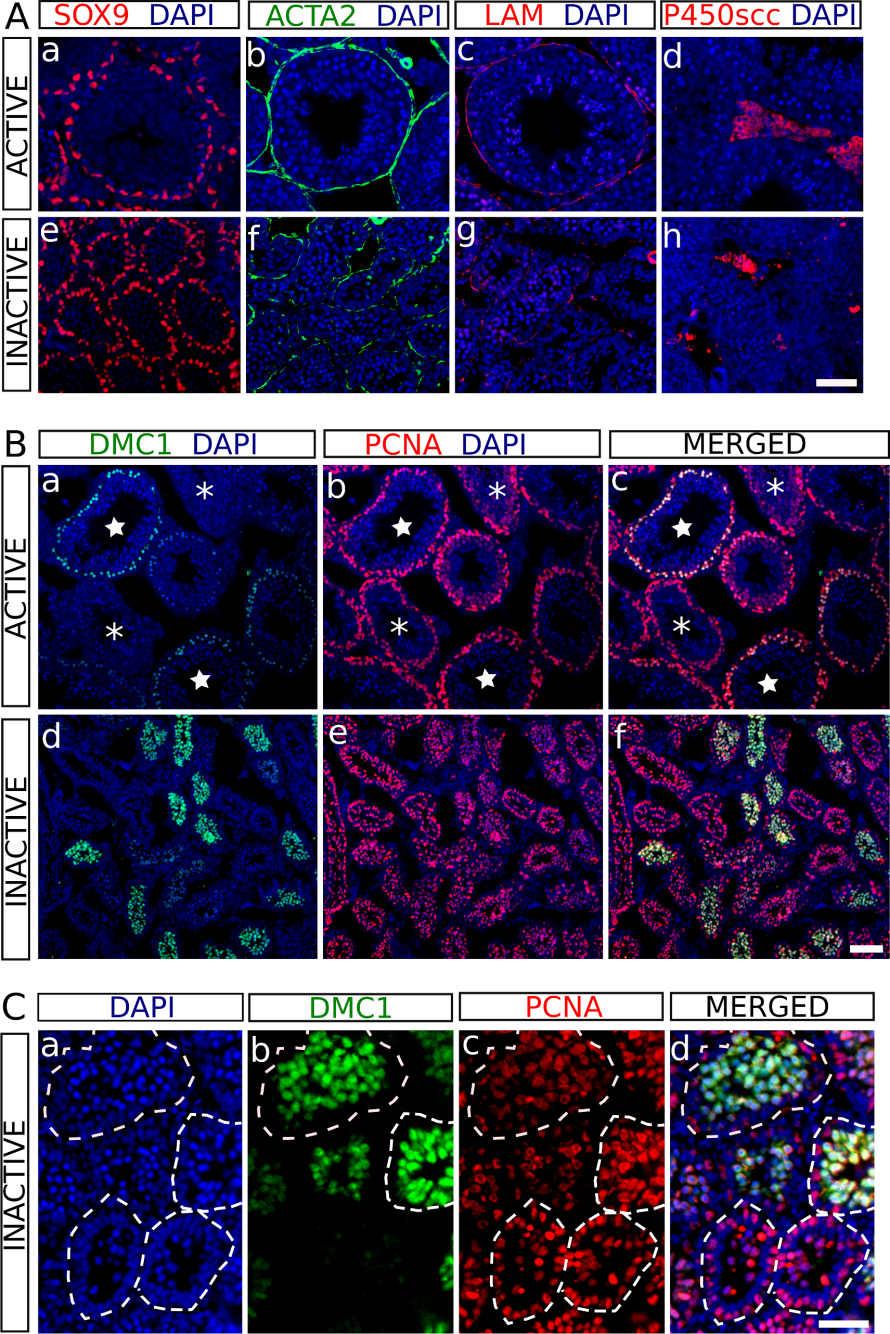
Immunofluorescence for several cell-type-specific molecular markers on histological sections of testes from *H*. *auritus*. A) Analysis of the somatic cell markers SOX9 (a, e), ACTA2 (b, f), LAM (c, g) and P450scc (d, h) in active (a-d) and inactive (e-h) testes. Sertoli cells (SOX9), peritubular myoid cells (ACTA2), basal lamina (LAM) and Leydig cells (P45scc) are identified with these protein markers. B) Double immunofluorescence for the germ cell markers DMC1 and PCNA in active (a-c) and inactive (d-f) testes. In active testes, DMC1^+^ cells (a) were only observed in leptotene-to-early pachytene spermatocytes of the seminiferous tubules at the spermatogenic stages VII-IX (stars). PCNA expression (b) was detected in mitotic spermatogonia as well as in zygotene and pachytene spermatocytes. Both proteins co-express in zygotene and early pachytene spermatocytes of tubules at stages VII‐IX (c; stars), but not in tubules containing later pachytene spermatocytes (asterisks). In inactive testes, both markers also showed a dynamic pattern of expression similar to that of active testes, but immunoreactive cells did not present a ring-like organization and appeared clustered together (d-f). C) Higher magnification of an inactive testis showing that the luminal region of the regressed tubules was filled with either DMC1^+^ PCNA^+^ (early pachytene) or DMC1^-^ PCNA^+^ (late pachytene) spermatocytes (dashed lines outline the tubular perimeter). Scale bar shown in Ah represents 50 μm for A, scale bar in Bf represents 100 μm in B and scale bar in Cd represents 50 μm in C.

Next, we studied the expression of somatic-cell-specific molecular markers by means of immunofluorescence in both summer (hereafter referred to as active) and winter (hereafter referred to as inactive) testes of *H*. *auritus*. SOX9 is a transcription factor necessary for adult Sertoli cell survival and function [19]. In the active testes, SOX9^+^ cells were evenly distributed at basal positions of the seminiferous tubules (**Fig 3Aa**). In the inactive testes, SOX9^+^ cells were also localized at the periphery of testis tubules; however, the distance between neighbouring SOX9^+^ cells was notably reduced when compared to that of active tubules, confirming that Sertoli cells in the regressed testes undergo a severe shrinkage (**Fig 3Ae**). We also analysed α-smooth muscle actin (ACTA2), which in the testis is a marker for peritubular myoid cells and arterial muscle fibres [20], and laminin (LAM), a principal component of the basement membrane [21]. In the active testes, we found a strong and moderate immunoreactivity for ACTA2 and LAM, respectively, surrounding the seminiferous tubules (**Fig 3Ab and 3Ac**). In the inactive testes, the staining for both ACTA2 and LAM, was weaker and irregular in shape, again, as a consequence of the involution of the seminiferous tubules (**Fig 3Af and 3Ag**). Finally, we also studied the expression pattern the cholesterol side-chain cleavage enzyme, P450scc, a protein involved in the synthesis of testosterone which is produced by Leydig cells. In both active and inactive testes, P450scc expression was clearly visible in the interstitial cells, indicating that Leydig cells of regressed testes maintain the steroidogenic function in some extent (**Fig 3Ad and 3Ah**).

We also studied the expression of germ cell-specific markers. DMC1 is present in leptotene-to-early pachytene primary spermatocytes [22] and the proliferating-cell nuclear antigen (PCNA) is expressed in spermatogonia as well as in zygotene and pachytene, but not leptotene spermatocytes [23]. Active testes exhibited both DMC1 and PCNA positive cells (**Fig 3Ba–3Bc**), which were located at the periphery of the seminiferous tubules showing an expression pattern that varied depending on the spermatogenic cycle stage, as described previously [6, 24]. DMC1^+^ cells were only observed in tubules of the spermatogenic stages VII-IX (stars in **Fig 3Ba**), whereas PCNA was expressed in proliferating spermatogonia as well as in a variable number of spermatocytes depending on the stage of the spermatogenic cycle (**Fig 3Bb**). Both proteins co-localized in zygotene and early pachytene spermatocytes of seminiferous tubules at stages VII-IX (stars in **Fig 3Bc**), but not in seminiferous tubules containing later spermatocytes (asterisks in **Fig 3Bc**). The inactive testis also showed a dynamic pattern of expression for both markers, although immunoreactive cells did not exhibit a ring-like organization and clustered together (**Fig 3Bd–3Bf**). At a higher magnification, we could observe that the centres of the testis tubules were completely filled with either DMC1^+^ PCNA^+^ (zygotene and early pachytene) or DMC1^-^ PCNA^+^ (later pachytene) spermatocytes (**Fig 3C**). Altogether, our results show that in the inactive testes of *H*. *auritus* meiosis onset is not halted but meiotic arrest occurs at the pachytene stage.

### Primary spermatocytes undergo apoptosis in the inactive testis of *Hemiechinus auritus*

**Fig 4:**
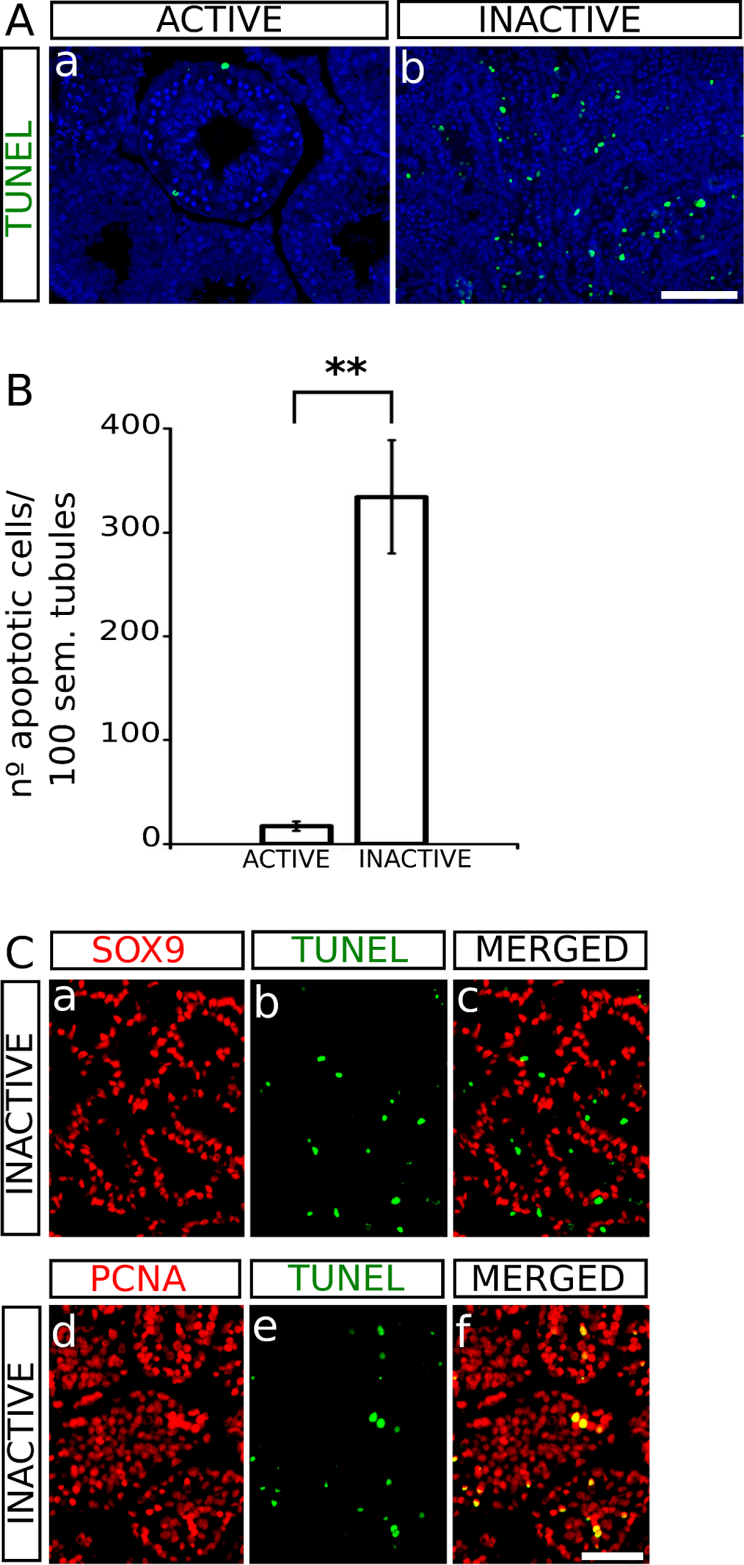
Study of apoptosis (TUNEL assay) in the testes of active and inactive males of *H*. *auritus*. A) The abundance of apoptotic cells (green) was clearly lower in active (a) than in inactive testes (b). B) Quantification of the incidence of apoptosis in active and inactive testes. C) (a-c) Double TUNEL-SOX9 immunofluorescence in inactive testes. Note that red (SOX9) and green (TUNEL) signals never colocalize, showing that Sertoli cells were not dying. (d-f) Double TUNEL-PCNA staining. All TUNEL^+^ cells also expressed PCNA (yellow cells in f), indicating that apoptotic cells are spermatocytes. Scale bar shown in Ab represents 100 μm for A and scale bar in Cf represents 50 μm in C.

Since apoptosis is a process normally occurring in the regressed of testes of seasonal breeding mammals [25], we performed TUNEL assay in both, active and inactive testes of *H*. *auritus*. Active testes only contained some few positive cells in a reduced number of seminiferous tubules, whereas the number of apoptotic cells was notably higher in inactive testes (active: 17 ± 4 apoptotic cells per 100 tubular sections; inactive 334 ± 54 apoptotic cells per 100 tubular sections; two-tailed *t*-test, P = 0.0094; **Fig 4A and 4B**). To identify the cell types dying in the inactive testes, we performed TUNEL assay together with immunofluorescence for cell type-specific markers. We observed that SOX9^+^ cells were never TUNEL^+^ (**Fig 4Ca–4Cc**), suggesting that Sertoli cells were not affected. In contrast, all TUNEL^+^ cells showed immunoreactivity for PCNA (yellow cells in **Fig 4Cf**), showing that an apoptosis-mediated massive loss of germ cells occurs in the inactive testes of *H*. *auritus*.

### Germ cell desquamation accounts for the massive germ cell loss occurring during the testicular regression of *Hemiechinus auritus*

**Fig 5:**
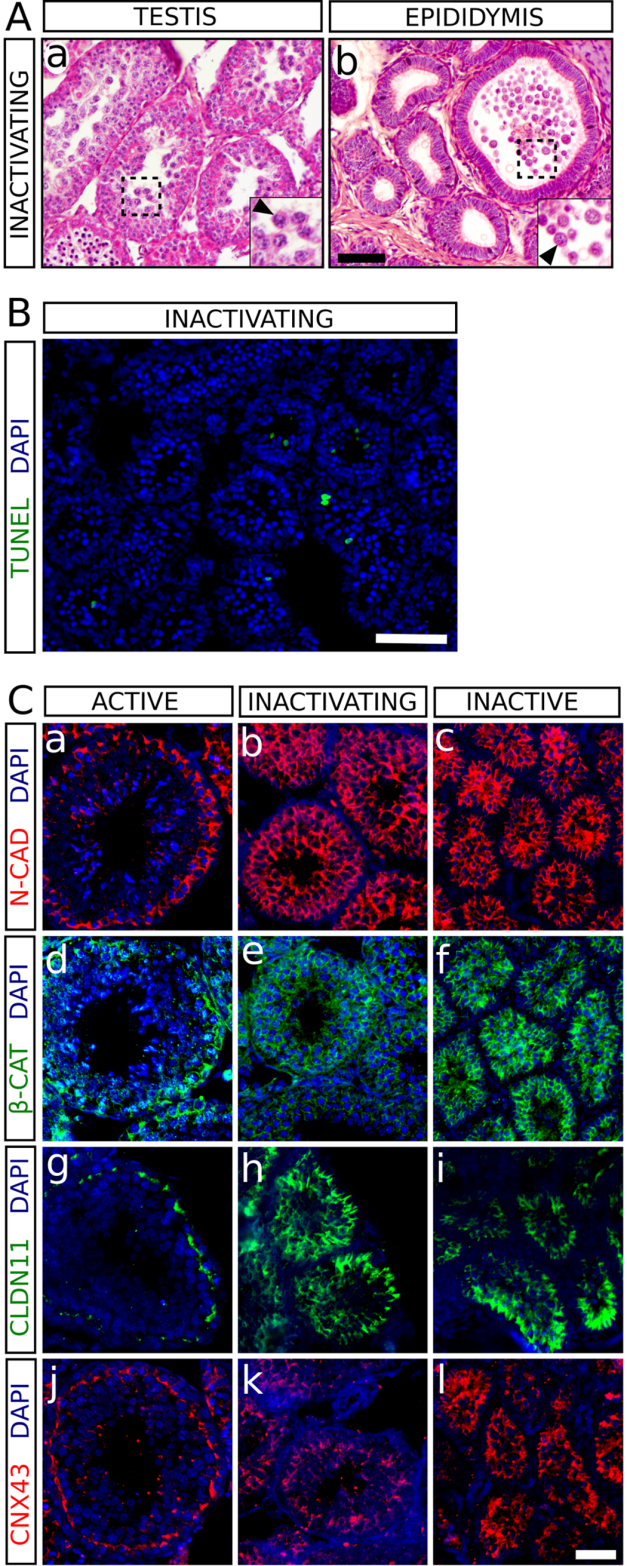
Study of the testes of *H*. *auritus* during the regression period. A) Hematoxylin and eosin staining of a histological section of an inactivating testis (a) and epididymis (b). During testis regression, seminiferous tubules exhibited a disorganized germinative epithelium, with primary and secondary spermatocytes occupying the lumen (see the inset in a). Round germ cells, which can be identified as primary spermatocytes (arrowhead in the inset in a), were also present in some sections of the epididymal tubule (arrowhead in the inset in b). B) TUNEL assay (green fluorescence) showing the location of several apoptotic cells. The luminal region of the seminiferous tubules in inactivating testes is mostly occupied by non-apoptotic cells. C) Expression of cell-adhesion molecules in active (a, d, g, j), inactivating (b, e, h, k) and inactive (c, f, i, l) testes. Immunofluorescence for N-CAD (a-c), β-CAT (d-f) CLDN11 (g-i) and CNX43 (j-l) showed that their expression pattern in inactive and inactivating testes is disorganized when compared with that of active testes. Scale bar shown in Ab represents 75 μm in A, scale bar in B represents 75 μm and scale bar in Cl represents 50 μm in C.

Two individuals captured at the beginning of Autumn (end of September) presented testes in which the process of regression was taking place (hereafter referred to as inactivating). In these testes, the mean diameter of the seminiferous tubules was in between those of the summer and the winter group (120 ± 12 μm). The germinative epithelium looked disorganised in many tubules and primary and secondary spermatocyte were anomalously located in the lumen (**Fig 5Aa**), showing that they were sloughed from their usual location in more basal regions. We also detected this type of germ cells within the lumen of the epididymal tubule (**Fig 5Ab**). We performed TUNEL assay to check whether apoptosis was responsible for the massive loss of germ cells that takes place in the regressing testes of the long-eared hedgehog. Dying cells were observed within testis cords of inactivating testes, although few of them were located at, or nearby, the lumen (**Fig 5B**), thus indicating that desquamation mainly affects living cells. These results suggest that, although apoptosis may contribute in some extent to the massive germ cell depletion affecting inactivating testes, additional mechanisms must be involved in the process. Two recent studies have shown that in the testes of two seasonal breeders, the Iberian mole *T*. *occidentalis* [10] and the large hairy armadillo *C*. *villosus* [11], the loss of Sertoli-germ cell junctions leads to a rapid desquamation of germ cells during the testis regression period. To determine whether the expression of proteins forming Sertoli-Sertoli and Sertoli-germ cell junctions was altered in the regressing testes of *H*. *auritus*, we performed immunofluorescence for several important adhesion molecules on histological sections of active, inactivating, and inactive testes. N-cadherin (NCAD) and β-catenin (β -CAT) are structural components of the adherens junctions as well as of the ectoplasmic specialization existing between Sertoli-Sertoli and Sertoli-germ cells [26]. Immunoreactivity for these two proteins in active testes was relatively stronger in the basal compartment than in the adluminal region, which showed a faint staining (**Fig 5Ca and 5Cd**). Claudin 11 (CLDN11) is a transmembrane protein of the tight junctions forming the blood-testis barrier established between adjacent Sertoli cells [27], and Connexin 43 (CNX43) is a component of the gap junctions found in Sertoli-Sertoli and Sertoli-germ cell contact areas [28]. In active testes, these two proteins were found in a spermatogenic cycle-dependent pattern, located mainly in the basal compartment of the germinal epithelium (**Fig 5Cg and 5Cj**). In contrast, in both inactivating and inactive testes, a homogeneous disorganized staining was observed for the four molecules throughout the entire germinative epithelium (**Fig 5Cb, 5Cc, 5Ce, 5Cf, 5Ch, 5Ci, 5Ck and 5Cl**). These results suggest that the adhesion between Sertoli cells and between Sertoli and germ cells is compromised in the regressing testes, thus explaining the germ cell sloughing taking place during this period.

### Serum testosterone levels are reduced in winter long-eared hedgehogs

**Fig 6:**
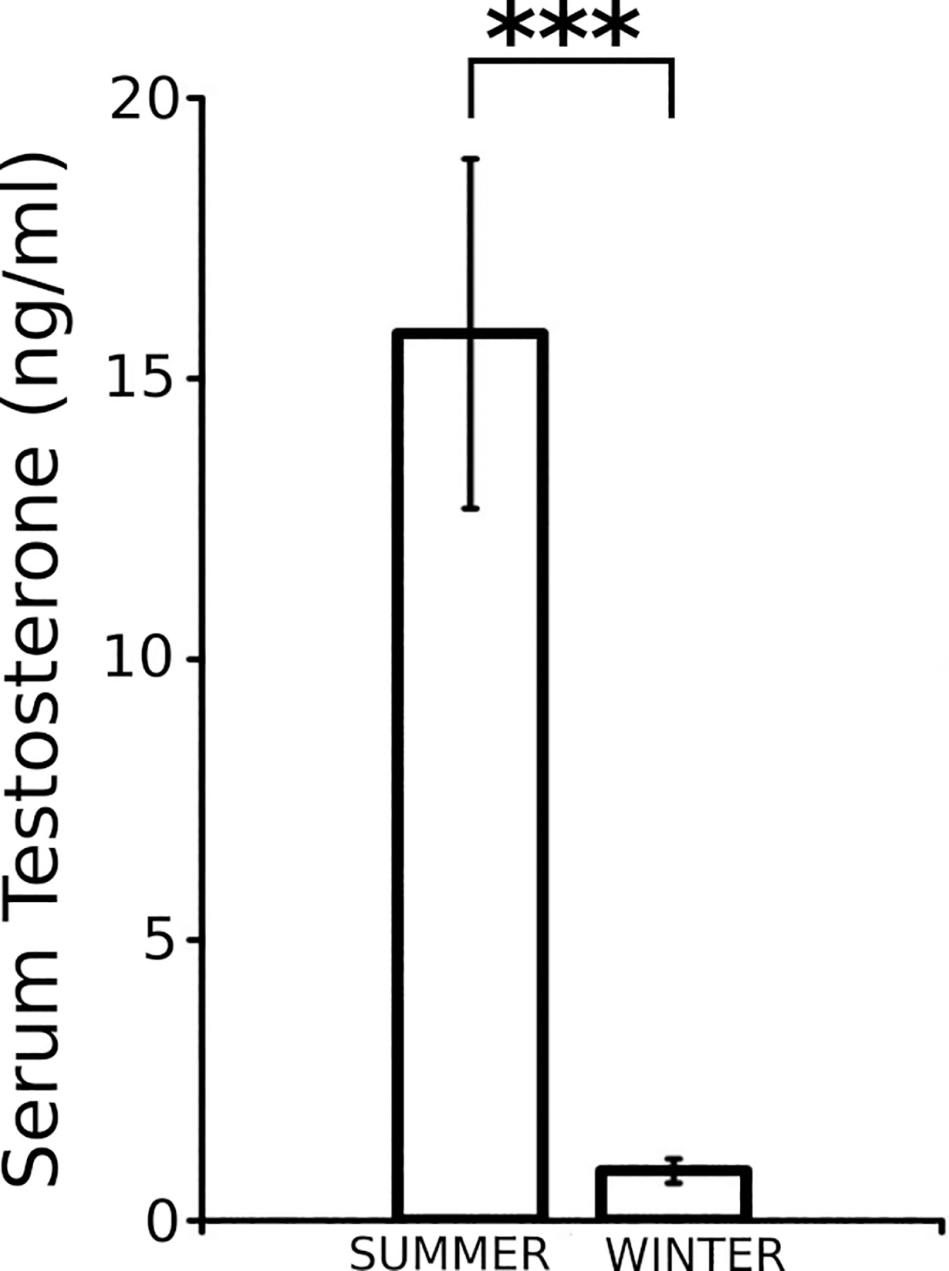
Serum testosterone concentrations in summer and winter males of *H*. *auritus*.

Reduction of the levels of serum testosterone in males during the non-reproductive season is a common feature of all seasonal breeding mammals studied to date. In the case of *H*. *auritus*, the mean levels of serum testosterone were significantly reduced in the individuals of the winter group when compared to those of the summer group (summer: 15.8 ± 3.11 ng/ml; winter 0.9 ± 0.21 ng/ml; two-tailed *t*-test, P < 0.001; **Fig 6**).

## Discussion

Testis regression is the process by which males of seasonally breeding species inactivate their gonads before they enter the resting period of the circannual reproductive cycle. This is necessary if the non-breeding period is long enough to make testis regression efficient in terms of energy saving, as spermatogenesis is too expensive to be maintained when it is not needed [6]. After the study of a number of seasonal breeding mammals, it seems evident that there is not a unique mechanism of testis regression, existing two main cellular processes by which germ cells are massively depleted: apoptosis and desquamation [7]. However, more species must be studied to determine their relative prevalence and, maybe, to discover new testis regression mechanisms. Here we report the first comprehensive study of the circannual changes affecting the testes in the long-eared hedgehog *H*. *auritus*. Our results show that in this species the main testis regression effector is germ-cell desquamation, as described previously in both the Iberian mole *T*. *occidentalis* [10] and the large hairy armadillo *C*. *villosus* [11, 29]. The presence of abundant primary spermatocytes (zygotene and pachytene) in the lumen of both seminiferous tubules and epididymides indicates that immature meiotic cells detach precociously from Sertoli cells, thus being sloughed and finally eliminated through the distal section of the genito-urinary tract. This is consistent with the disorganized distribution of the cell-adhesion molecules that we observed in the seminiferous tubules of both inactivating and inactive testes of *H*. *auritus*.

Apoptosis is an essential process occurring normally in functional fertile testes. It facilitates the regulation of the spermatogonial population density and safeguards the genetic integrity of the male gamete by eliminating cells unable to pass checkpoint transitions derived from improper meiotic chromosome synapsis [9, 30–33]. Also, apoptosis has been shown to be the cellular process responsible for seasonal testis regression in many tetrapod species, including some mammals [2, 9, 25, 34–38]. However, like in the roe deer *Capreolus capreolus* [39], the Iberian mole *T*. *occidentalis* [4], and the large hairy armadillo *C*. *villosus* [11], we found that apoptosis is not the primary cause of germ-cell depletion in the regressing testis of *H*. *auritus*. The low abundance of apoptotic cells in regressing testes supports this finding. Nevertheless, as in the mole, apoptosis plays an important role in the inactive testes of *H*. *auritus*, by eliminating all primary spermatocytes that reach the pachytene stage.

This process of apoptosis-mediated spermatocyte depletion is needed because meiosis onset is not halted in the inactive testis of this hedgehog species. The presence of both seminiferous tubules containing DMC1^+^ cells and others lacking them in inactive testes (like in active ones), a finding also described in the Iberian mole [24], suggests that the rhythm of meiosis entry, that determines the timing of the spermatogenic cycle, is maintained in the testes of inactive males of these species. This, in turn, implies that the mechanisms controlling meiosis initiation remain functional in inactive testes. Alternatively, males of other species that completely stop the meiosis onset, show testes containing just Sertoli and spermatogonial cells during the non-breeding season. The latter strategy is far more efficient and inexpensive to maintain testes sterile during the resting period, and more species in which non-breeding males stop completely the meiosis entry would be expected to exist. Paradoxically, the former strategy is more frequent in tetrapods. For instance, meiosis onset is maintained in the inactive testes of the Japanese red-bellied newt *Cynops pyrrhogaster* [40], the silver fox *Vulpes vulpes* [41], the Syrian hamster *Mesocricetus auratus* [2, 37], the Chinese soft-shelled turtle *Pelodiscus sinensis* [42], and the Japanese jungle crow *Corvus macrorhynchos* [43], as well as in the Mediterranean pine vole *Microtus duodecimcostatus* (our unpublished results), and the long-eared hedgehog *H*. *auritus* (present paper), amongst others. On the other hand, complete inhibition of meiosis entry occurs in the inactive males of the white-footed mouse *Peromyscus leucopus* [44], the European starling *Sturnus vulgaris* [25], and the large hairy armadillo *Chaetophractus villosus* [11, 29]. The presence of both strategies in at least two classes (mammals and birds) of the superclass Tetrapoda, as well as in different clades inside these classes, suggests that both processes may have appeared repeatedly during the evolution of these taxa. According to the Wright’s theory on fitness landscapes [45], species adopting the complete meiotic inhibition strategy would be occupying an adaptive peak higher than that of species retaining some meiotic activity, but the later species could not benefit from the other (better) strategy, passing from one peak to the other, because this would require a transient loss of fitness. This would explain the persistence of so many species that retain some meiotic activity during the non-breeding season.

A common feature of seasonal testis regression is the permeation of the blood-testis barrier (BTB), a specialised junctional complex that defines an adluminal and a basal compartment in the germinative epithelium and preserves meiotic and post-meiotic germ cells (located in the adluminal compartment) from the action of the immune system [46, 47]. Seasonal changes in the BTB permeability have been tested by molecular-tracer experiments in three mammalian species: the mink *Mustela vison* [48], the Djungarian hamster *Phodopus sungorus* [49], and the Iberian mole *T*. *occidentalis* [10]. In all of them, it was shown that the BTB loses its normal impermeability during the testis regression process. We could not perform *in vivo* molecular tracing experiments with the hedgehogs included in this study, but the disorganised expression pattern observed for CLDN11 in the testes of inactive males of this species clearly resemble what we previously described in the mole, and suggests that the BTB of the *H*. *auritus* testis also becomes permeable during seasonal regression.

Subsequent to the identification of germ cell desquamation as the principal testis regression effector in the Iberian mole *T*. *occidentalis* [10], this mechanism was also described in the large hairy armadillo *C*. *villosus* [11, 29]. Here we report that this cellular process also operates in the long-eared hedgehog *H*. *auritus*, thus suggesting that it could be relatively common in mammals. The testis regression process of this hedgehog species is similar to that described for the mole, as it includes 1) massive desquamation of meiotic and post-meiotic germ cells, 2) irrelevance of apoptosis during regression, 3) disorganised expression of cell-adhesion molecules, 4) persistent meiosis onset, 5) apoptosis of pachytene spermatocytes, and 6) probable BTB permeation. The only noticeable difference between these two species concerns the re-organization of the interstitial tissue during testis regression. In the mole, Leydig cells form a dense, continuous cell matrix that occupies most of the inactive testis volume [10], a phenomenon not observed in the hedgehog. On the other hand, the armadillo shows some significant differences with respect to both the mole and the hedgehog. Three main features were characteristic in the armadillo but not in the other two species: 1) desquamated cells die subsequently by apoptosis, 2) Sertoli cells phagocytose residual germ cells in the latest stages of regression, and3) meiosis onset is completely abolished in the inactive testis. Referring to the few mammalian species studied before 2013, we suggested a possible phylogenetic origin for the inter-species differences reported in the process of testis regression, as species with apoptotic-based mechanisms (the Syrian hamster *M*. *auratus* [38]) belong to the superorder Euarchontoglires, whereas those with testis regression not based on apoptosis (the roe deer *C*. *capreolus* [39] and the Iberian mole *T*. *occidentalis* [4]) belong to the superorder Laurasiateria [10]. Further evidences indicate that both the large hairy armadillo [11] and the long-eared hedgehog (present paper), like the mole [10], present a germ-cell desquamation-based testis regression process, and also belong to the superorder Laurasiateria. Moreover, both the mole and the hedgehog have a very similar mechanism of testis regression and belong to the order Eulipotyphla, whereas the armadillo, which exhibits more differences in this respect, belongs to the order Cingulata. Hence, current data support a phylogenetic origin for the inter-clade differences observed in the process of testis regression and suggest that a high variation rate probably exists for this process. The study of a higher number of species is needed to shed light on this issue.
